# Japanese Systems to Support Inclusive Education for Children Requiring Medical Care, Current Status of Such Support, and Related Challenges—Based on the Results of Surveys Involving Departments of Education Supporting their Enrollment in General Schools

**DOI:** 10.3390/children6030039

**Published:** 2019-03-04

**Authors:** Tomoko Yamamoto, Koichi Moriwaki

**Affiliations:** 1 Department of Child Development, Faculty of Humanities, Saitama Gakuen University, Kawaguchi 3330831, Japan; 2 Department of Pediatrics, Saitama Medical Center, Saitama Medical University, Kawagoe 3508550, Japan; kmoriwa@saitama-med.ac.jp

**Keywords:** inclusive education, children requiring medical care, municipal departments of education, general schools, Japan

## Abstract

Based on the results of our surveys involving several municipal departments of education that support the enrollment of children requiring medical care in general schools, this report describes Japanese systems to support inclusive education for these children, the current status of such support, and related challenges. The municipal departments of education that systematically support inclusive education for children requiring medical care began to promote such education in their communities before the initiation of nationwide enrollment support for the children. In addition, their sections in charge of enrollment support also manage affairs related to human rights education, rather than general affairs for special support and education. Such a variation in the sections in charge resulted in differences in the purposes of support. Further nationwide surveys should be conducted to develop universal design principles and comprehensively support inclusive education for children requiring medical care.

## 1. Introduction 

In Japan, the number of births is decreasing, whereas the proportion of children requiring medical care is increasing. The Japanese Society for Pediatric Home Care Support reported that the number of children aged 19 or younger requiring medical care reached 17,209 in FY (Financial Year. from April 1 to March 31 in Japan.) 2015, which is almost double the 9403 in FY2005 [1]. Japanese advanced technologies and systems for neonatal care are background factors of this increase. 

Children requiring medical care need to use medical devices for tube feeding, tracheostomy care, or other purposes. Many children requiring medical care also have severe physical and intellectual disabilities. It is necessary to use stretchers and wheelchairs customized for individual children, and to adjust equipment to maintain their posture. However, some children requiring medical care have no intellectual disability and walk to general schools in the neighborhood, although they require the use of ventilators or other medical equipment [2]. In Japan, these children are admitted to general or special support schools. In this report, public schools excluding special support schools are referred to as “general schools”. Special support schools are classified based on the types of disability: Visual, auditory, and intellectual disabilities, impairment of the arms and legs, or being physically weak/fragile. There are 1135 special support schools in Japan, and 71,802 students have been admitted to special support elementary and junior high schools [3]. Some disabled students who wish to advance to general schools are advised to attend special support classes at general schools or take classes spanning multiple school years. There are 858 children requiring medical care enrolled in public elementary and junior high schools in Japan [4]. 

In 2011, when the Basic Act for Persons with Disabilities was revised, the following description was added to Article 16: “Necessary measures, such as improving and developing the content and methods of education, should be implemented, while making arrangements for all school-age children with and without disabilities to learn together as much as possible”. 

In the following year, 2012, the Central Council for Education, which is an advisory body for the Ministry of Education, Culture, Sports, Science, and Technology in charge of education-related administration, published a report entitled: Promotion of Special Support and Education to Establish Inclusive Education Systems toward the Realization of a Collaborative Society. Focusing on collaborative learning among preschoolers, school-age children, and students with special educational needs, the report emphasized the importance of creating a diverse and flexible framework to provide education that most appropriately accommodates the needs of these children with insight into their future independence and social participation. 

Furthermore, the Act for Eliminating Discrimination against Persons with Disabilities was enacted in 2013, with the following Article 1: Purpose: “To realize a collaborative society that equally allows all people with and without disabilities to live together based on mutual respect for personality and individuality”. 

In 2014, when the United Nations established the Convention on the Rights of Persons with Disabilities, Japan agreed to the following approaches specified in Article 24: “State parties recognize the right of persons with disabilities to education”, and therefore, “with a view to realize this right without discrimination and on the basis of equal opportunity”, “they shall ensure an inclusive education system at all levels”. As another measure to ensure such a right, “State parties shall ensure that reasonable accommodation of the individual’s requirements is provided”. 

Furthermore, in 2018, the United Nations Committee on the Rights of the Child presented some questions to Japan as a ratification of the Convention on the Rights of the Child after receiving its report [5]. Through these questions, the Committee requested that Japan provides information regarding the progress of inclusive education for children with disabilities, such as the number of those belonging to special support and general schools. 

With the importance placed on the realization of inclusive education for children with disabilities as a national and international trend, the numbers of children requiring medical care and of those enrolled in general schools are increasing in Japan. A previous study on support for the enrollment of children requiring medical care in general schools examined school nurses’ recognition of support for these children to enhance their sociability, and reported that the nurses recognized the necessity of providing enrollment support based on the children’s and their parents’ actual needs [6]. In such situations, it may be important to clarify current systems to support the enrollment of children requiring medical care in general schools as needed, measures to promote liaison and collaboration between teachers and medical professionals, such as nurses, and the status of such liaisons and collaborations. Therefore, this report discusses the characteristics of such support and the related challenges based on the results of our interview surveys involving municipal departments of education that support the enrollment of children requiring medical care in community schools to examine their support systems and the status of support. 

## 2. Materials and Methods

In the following section, the results of our interview surveys involving municipal departments of education that support the enrollment of children requiring medical care in general schools to investigate their systems and the current status of support are presented. As the present survey focused on systems that have significant effects on the inclusion of children requiring medical care, it was conducted in cooperation with the educational boards across Japan that play key roles in the establishment of these systems. Based on the results, the characteristics of enrollment support provided by municipal departments of education and related challenges were clarified. 

Among the municipalities of Japan that are establishing systems to support the enrollment of children requiring medical care in general schools, we conducted interviews with the departments of education of Yokohama City and Toyonaka City, which consented to cooperate. We have added the explanation that these two cities were selected as the subjects of the survey because they have developed systems to encourage general schools to accept children requiring medical care and agreed to cooperate with the survey. Toyonaka City in particular is one of the two non-ordinance-designated cities that have been promoting the establishment of such systems, drawing attention from around Japan.

We interviewed 2 employees of the Yokohama City Department of Education in May 2018. During the interviews, we presented questions regarding the city’s systems to support the enrollment of children requiring medical care in general schools and the status of such support. The employees responded to these questions in a free-description style. Similarly, we interviewed 1 employee of Toyonaka City at a municipal education center in October 2018. During the interview, we presented questions regarding the city’s systems to support the enrollment of children requiring medical care in general schools and the status of such support, and the employee responded to these questions in a free-description style.

Regarding basic ethical considerations, we adopted the following measures when conducting the previous surveys: Ensuring human rights, respecting each person’s dignity and free will, protecting personal privacy, confirming the appropriateness and rationality of the study content and procedures, and avoiding possible disadvantages and risks related to the surveys.

Additionally, we considered the following items when planning and implementing the surveys: Planning the procedure and method to ask candidate institutions to cooperate with the study clearly and in detail, asking candidate institutions to only cooperate with processes that are necessary and indispensable, avoiding the forcing of candidate institutions to cooperate with the surveys, allowing candidate institutions not to consent to participate in any or some processes of the surveys and to withdraw their consent at any time, providing psychological considerations to candidate institutions, and preventing the identification of individuals other than candidate institutions.

We examined the Department of Education of Yokohama City through interviews with 2 city officials in May 2018. During the interviews, we mainly discussed the city’s systems to support the enrollment of children requiring medical care in schools and the current status of such support.

In addition, we investigated the Department of Education of Toyonaka City at a municipal education center in October 2018. We mainly interviewed 1 city official. During the interview, we mainly discussed the city’s systems to support the enrollment of children requiring medical care in general schools and the current status of such support.

## 3. Results

### 3.1. Yokoyama City

**Figure 1:**
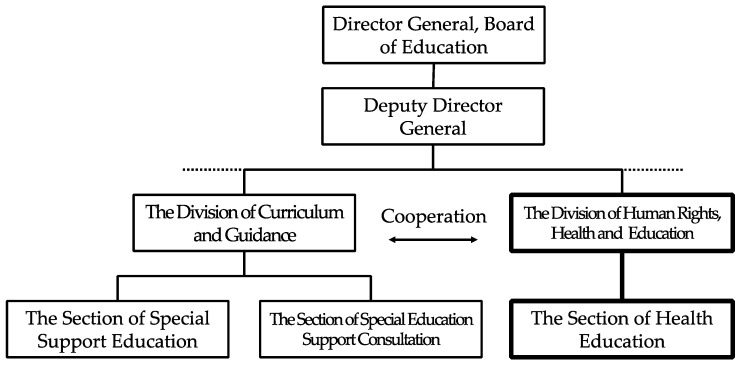
Organization of Yokohama City Board of Education (Excerpt).

Yokohama City is the prefectural office of Kanagawa Prefecture. It is one of the ordinance-designated cites and has administrative districts in 18 wards. The current population is approximately 3.7 million. The number of elementary and junior high schools, and the number of children and students are the highest among the ordinance-designated cites. The information on inclusive education for children requiring medical care is listed below in the Figure 1.

As can be seen from above figure, in Yokohama City, the Section of Health Support, Division of Human Rights, Division of Human Rights, Health, and Education, Secretariat of the Departments of Education, is in charge of school enrollment support. Although the Section of Health Education plays a major role in supporting enrollment in general schools, it cooperates with the Section of Special Support Education and the Section of Special Education Support Consultation as necessary.

Until the present, it has provided such support for 1 child in FY2017 and 2 children (1 of them is still being supported) in general schools; in all cases, support started from enrollment. At present, support is being provided for first- and second-grade elementary school students, including a child who has been supported since enrolling in a private kindergarten accepting children requiring medical care.

As shown above Table 1 below, there are 12 special support schools in Yokohama City, and 1528 students were enrolled in elementary schools or junior high schools in FY2018. On the other hand, in Yokohama City, there are three school types as general schools, including two compulsory education schools and combined elementary and junior high schools.

Yokohama City defines support for these children as follows: “giving considerations for students requiring daily medical care at school, and supporting the elementary and junior high schools they belong to through medical care provided by nurses at these schools”.

It is based on the following criteria: Among all students of elementary and junior high schools providing compulsory education in Yokohama City, those requiring daily medical care at school; those regarded as being able to attend general schools, but requiring medical care at school by their doctors, with their parents understanding the relevant support program and requesting medical care provided by nurses, and the principal and school committee agreeing with the provision of such care; and those defined as children requiring medical care at the Medical Care Coordination Meeting from specialized perspectives. The necessity of support is re-examined annually at the Medical Care Coordination Meeting.

Care procedures performed at school include: Suctioning sputum in the oral cavity and suctioning rhinorrhea in the nasal cavity, suctioning sputum in tracheal cannulas, gastro- or enterostomy tube feeding, and nasal tube feeding. Among these, the city covers suctioning sputum in the oral cavity (not including the management of artificial respiration or oxygen therapy in any case), suctioning rhinorrhea in the nasal cavity, and suctioning sputum in tracheal cannulas. Depending on the operational situation, the scopes of suctioning rhinorrhea in the nasal cavity and suctioning sputum in tracheal cannulas may be expanded in the future.

Schools can be equipped with medical devices, but their management, such as cleaning, replacement, and replenishment, should be performed by parents. Yokohama City also provides counseling for parents. The principal of each school confirms the conditions of the relevant children and their parents during health examinations on admission. When a school has decided to support a child, it sends an application to the Section of Health Education at the request of the child’s parents. Based on this application, the Section of Health Education holds a Medical Care Coordination Meeting to examine the necessity of support. Medical Care Coordination Meetings are held with the Section of Health Education and Section of Special Support Education as secretariats. Representative school doctors (medical association), principals (principals’ association), nurse-teachers, and those involved participate in the meetings to discuss approaches to care, and the details of the equipment and personnel support, and develop future perspectives on medical care in Yokoyama City. When they decide to support the child, with methods to support him or her determined, the Section of Health Education forms contracts with home-visit nursing facilities belonging to the medical association. These facilities are closed on weekends and national holidays, in principle, but their services may also be available on these days (e.g., during an educational school trip) depending on the agreement.

In addition to the above-listed approaches, the Section of Special Education Support Consultation provides school enrollment counseling for parents as necessary. The section provides and shares information with relevant schools, and adopts actions based on the results of examinations. It also shares information and collaborates with the Section of Health Education.

### 3.2. Toyonaka City

Toyonaka City is located in Osaka Prefecture. It is one of the cabinet order-designated core cities with a population close to 400,000 people and public health centers.

Toyonaka City has implemented innovative educational approaches toward “collaborative learning and collaborative growth”, expanding the activities of the parents of children with disabilities, groups supporting such children, community residents, and teachers since 1978, when Toyonaka City’s Basic Policy on Education for Children with Disabilities was established [8]. “Collaborative learning and collaborative growth” is also one of the principles of education adopted by Osaka Prefecture [9]. Toyonaka City’s Basic Policy on Education for Children with Disabilities was revised in 2016. Based on this revised version, the city is currently supporting the enrollment of children requiring medical care in general schools. It also established Toyonaka City’s Basic Policy on Early Childhood Education and Care for Children with Disabilities in the area of preschool education, which was revised in 2015. Integrated early childhood education and care is currently being provided based on the revised version in the city [10].

Adopting the “collaborative learning and collaborative growth” principle, Toyonaka City respects children’s and their parents’ desires as a prerequisite to decide schools. As of 1998, there were children requiring tube feeding who attended general elementary schools with support from their parents and supporters (rather than medical professionals, such as nurses, at that time). Around 2001, caregivers with a teaching license began to provide daily life support for children with emotional disorders and intellectual disabilities. Today, these children’s and their parents’ reasons for desiring general school enrollment are diversified from “to grow with healthy children at the same age” to “to learn at a general school close to home” and “to belong to the same school as their siblings”.

In Toyonaka City and all other areas in Osaka Prefecture, human rights education has been a background factor of inclusive education and care for children requiring medical care. In these areas, human rights education has been provided to eliminate discrimination against children with disabilities at school, while addressing human rights issues due to social discrimination against specific communities (“Dowa” problem), people of other nationalities living in Japan, and newcomers. In such educational and social environments, with public opinions represented by the “equality is the major premise” policy, the city has examined practical measures to promote the enrollment of children requiring medical care in general schools.

Toyonaka City’s Basic Policy on Education for Children with Disabilities pursues inclusive education, with the goals of “establishing a collaborative society” and “maximizing the capabilities of children with disabilities”. This policy expects that children growing up under the inclusive education will lead to a more collaborative society. To promote inclusive education, as specified in this policy, the city has adopted a number of basic measures listed below, in addition to numerous commitments to schools, communities, society, children with disabilities, and their parents. The details of these basic measures are as follows:

(1) Education/enrollment counseling
  Establishing systems to provide specialized education counseling and support for children with disabilities from infancy through liaison with related institutions.
  Providing information regarding pre-enrollment processes, systems to determine schools, and counseling after the determination in the early stages.
(2) Determination of schools
  Providing sufficient information regarding elementary/junior high schools and special support schools within the school district of the community, with attentive enrollment counseling.
  Enrolling children with disabilities in elementary/junior high schools within the school district of the community while respecting their and their parents’ desires as much as possible, selecting the optimal schools for them, adopting opinions from experts, and considering the situation of the school/community.
(3) Fundamental environmental arrangements/reasonable accommodation—making fundamental environmental arrangements for school-age children/students with disabilities to receive high-quality education.
  Ensuring that reasonable accommodations for individual children are provided according to their conditions and educational needs, and based on equal opportunities for education.
(4) Personalized guidance to fulfill individual children’s needs
  Clarifying an individual child’s educational needs, and providing personalized guidance to fulfill such needs by utilizing individualized educational support and teaching plans.
  Providing guidance that fulfills an individual child’s educational needs by flexibly providing education, adopting varying teaching styles, and promoting appropriate support.
(5) School-wide expertise acquisition
  Enhancing teachers’ expertise in support and education through training programs.
  Effectively using external human resources to accommodate the diverse educational needs of school-age children/students with disabilities, and creating opportunities for school-wide expertise acquisition.
(6) Liaison with related institutions and seamless support
  Promoting liaison between schools and external institutions providing medical/welfare services to support the community lives of school-age children/students with disabilities.
  Sharing the details of support with children and their parents, and promoting liaison within related institutions from nursery schools, kindergartens, and certified preschools to elementary/junior high schools, developmental support centers, and support schools for school-age children to provide seamless, comprehensive support covering pre-enrollment processes to the determination of a career path after the completion of compulsory education.

The status of the school enrollment of children requiring medical care in Toyonaka City in FY2018 is shown below in the Table 2.

As indicated in above Table 2, there are two special support schools in Toyonaka City, and 266 students were enrolled in elementary schools or junior high schools in FY2018. On the other hand, in Toyonaka City, there are two school types as general schools.

In Toyonaka City, children requiring medical care enrolled in general schools mostly belong to general classes at school. To support the enrollment of these children in general schools, the city assigns eight teachers, including non-regular lecturers, to special support classes based on the type of disability, such as visual impairment, hearing impairment, intellectual disabilities, physical disabilities, and medically fragile conditions. Teachers in charge of special support classes attend all classes that the children requiring medical care attend, and support their learning with the teacher in charge of each class. Some of these children also individually learn in private rooms, but those whose parents place importance on their learning achievements often advance to special support schools with a larger number of teachers. In some cases, budgets to install special toilets and elevators are insufficient, consequently forcing children requiring medical care to attend school under the existing conditions.

The process of enrolling these children proceeds with enrollment counseling, as in other cities [14,15]. Enrollment counseling targets the parents of children requiring special educational considerations in school life due to impaired mental and/or physical development. The parents’ desire to receive enrollment counseling is sent from the nursery schools their children attend, for example, to the Education Support Group, Section of School-age child/Student Support within the municipal department of education. If their children do not belong to any school or they are living in other prefectures/cities, the parents need to directly contact this section. Enrollment counseling generally starts in June. After scheduling for individual parents and nursery schools, counseling sessions are held at these nursery schools or education centers. After counseling, arrangements are made for parents to observe general and/or special support schools, and receive consultation there. Subsequently, around mid-October, they receive an enrollment notification and information regarding health examinations for enrolled students from the Section of School Education. Their desire is reconfirmed before 30 November. Parents themselves also need to contact the Education Support Group, Section of School-age child/Student Support for such a reconfirmation. When they wish to enroll their children in special support schools, they should also contact the group before November 30. It should be noted that in Toyonaka City as a hub city, all employees of medical institutions, such as public health centers and municipal hospitals, and of the department of education are city officials, and this facilitates the sharing of information related to the enrollment of children requiring medical care among them prior to enrollment counseling.

In the case of children requiring medical care who desire to advance to general schools, their parents need to submit a letter of request to the head of the municipal department of education [16]. With the submission of this letter, a review meeting on the provision of medical care is held for each child, his/her parents, the person in charge from the municipal department of education, the principal of the relevant school, the supervisor engaged in elementary/junior high school education, nurses, and the head of the pediatrics department of the relevant municipal hospital to participate. The outcomes of the meeting are summarized in a report created by the head of the Toyonaka Municipal Hospital Department of Pediatrics, which is submitted to the regular doctor after being confirmed by the parents. This reporting process is performed by the parents, person in charge from the municipal department of education, principal of the relevant school, and nurses when vising the doctor. Having received the report, the regular doctor prepares an instruction form by the day of admission.

To provide medical care at school based on this instruction form, the parents are asked to accompany their child during the post-admission period between April and May, at least until the initiation of the school meal provision, and share appropriate medical care procedures for the child with nurses. The nurses who have learned the procedures create an operation manual, and submit it to the doctor in charge and the parents for confirmation. Assistance during daily school attendance, such as positioning and diaper changing, is also provided by teachers in charge of special support classes.

## 4. Discussion

The results of these surveys revealed two characteristics of the support for the enrollment of children requiring medical care in general schools provided by Japanese municipal departments of education.

First, the circumstances that led to the establishment of support systems varied between the two municipalities.

In Toyonaka City, which has implemented innovative educational approaches toward “collaborative learning and collaborative growth” since 1978, inclusive education for children requiring medical care has expanded on a community-wide basis based on the intentions of residents, including those not directly involved in such education. Under these circumstances, the city has implemented practical measures to ensure “equality as the major premise”. For example, special support teachers are also allocated to general schools based on the type of disability, and teachers in charge of special support classes participate in training programs to learn about medical care. While adopting these measures, Toyonaka City also established the Basic Policy on Early Childhood Education and Care for Children with Disabilities. Thus, it has actively developed systems to provide comprehensive education from the preschool period independently of the national school education system. The city also regards support for the determination of optimal schools for individual children requiring medical care, complying with the school education system, as one of its diverse approaches, represented by personalized guidance to fulfill each child’s educational needs, liaison with related institutions, and seamless support. The city adopts these approaches from the perspective of promoting human rights education. In addition to children with diseases and disabilities, including those requiring medical care, children of other nationalities living in Japan are supported through community-wide approaches to address social challenges influencing their development and learning. The presence of municipal public health centers and medical institutions, which start to support children requiring medical care when they are preschoolers, has also facilitated liaison and collaboration from the early stages as community-based resources available from the department of education.

Second, the section providing enrollment support also varied between the two municipalities. In Toyonaka City, the section directly supporting inclusive education for children requiring medical care is the Education Support Group, Section of School-age child/Student Support. In contrast, in Yokohama City, the Section of Health Support, Division of Human Rights, Health, and Education, Secretariat of the Departments of Education is in charge of school enrollment support. When providing school enrollment counseling for parents, the Section of Special Education Support Consultation shares related information and collaborates with the Section of Health Education as necessary.

Toyonaka City has organized its support systems based on the principle of human rights education, whereas the Division of Human Rights, Health, and Education directly provides enrollment support for children requiring medical care in Yokoyama City. In many other Japanese municipalities, sections providing enrollment support for these children are often in charge of special support/education or special support schools. Therefore, the case of Yokoyama City, where a division managing educational affairs related to human rights provides enrollment support, is an exception. We were unable to clarify the circumstances that led this division to directly support the enrollment of children requiring medical care, but the person currently in charge stated that it occurred too long ago to recall the situation. Furthermore, municipal departments of education that systematically support the enrollment of children requiring medical care as a human rights-related measure promote inclusive education for these children more actively.

Based on these findings, support for the enrollment of children requiring medical care may be characterized by being based on the idea of human rights as a background factor, and being systematically provided as a human rights-related measure. To implement such measures, the municipalities have established comprehensive systems for not only school education, but also preschool education, and to promote early liaison and collaboration with resources available in each community. They have also organized systems to support teachers, such as allocating special support teachers to general schools based on the type of disability, and providing training programs for teachers in charge of special support classes to learn about medical care.

On the other hand, systems to support the enrollment of children requiring medical care from the perspective of children’s rights protection have only been established in some communities, and universal design principles have yet to be put into practice. To address these areas, it may be necessary to clarify challenges and appropriate measures based on the results of nationwide status surveys.

Although local governments give us limited authority over making decisions on schools for children requiring medical care, the exercise of such an authority requires environmental arrangements, including personnel. Therefore, the long-term implementation of this policy is difficult without financial support from the central government. The Ministry of Education, Culture, Sports, Science, and Technology has been providing financial support to deploy nurses who can provide medical care at schools and promoting national surveys by local educational boards for inclusive education. It is necessary to develop/implement systems for collaboration and cooperation with parents, teachers, school nurses, and other people who directly support the children, in addition to continuous financial support from the central government.

## 5. Conclusions

Our interview surveys revealed differences in the circumstances that led to the establishment of support systems and the section in charge between the two municipalities as characteristics of enrollment support for children requiring medical care provided by the departments of education. On the other hand, human rights were the basis for the principles adopted by both municipalities. Moreover, the departments of education that systematically support the enrollment of children requiring medical care as a human rights-related measure promote inclusive education for these children more actively. The establishment of these systems should not be limited to some communities. As their further development based on universal design principles is essential, it may be necessary to identify challenges and determine appropriate measures based on the results of nationwide status surveys on the current systems and situations.

To effectively support inclusive education for children requiring medical care, it may be necessary to clarify challenges and appropriate measures based on the results of nationwide status surveys, and further develop universal design principles.

## Figures and Tables

**Table 1:** Yokohama City [7].

	Number of Schools		Number of Students	
School Type	Total	Schools for Students Requiring Medical Care	Total	Students Requiring Medical Care
Special Support School	12	12	1528	3
Elementary School	34	34	179,843	4
Junior High School	20	20	76,874	0
Combined E.S. and J.H.S.	2	2	1524	0
Postponement or Exemption of Schooling				0

**Table 2:** Toyonaka City [11,12,13].

	Number of Schools		Number of Students	
School Type	Total	Schools for Students Requiring Medical Care	Total	Students Requiring Medical Care
Special Support School	2	2	266	10
Elementary School	41	41	21,846	6
Junior High School	19	19	9447	2
Postponement or Exemption of Schooling				0
